# Supplementary material for: Peptides from the Sea Anemone Metridium senile with Modified Inhibitor Cystine Knot (ICK) Fold Inhibit Nicotinic Acetylcholine Receptors
Source: Toxins (Basel). 2022 Dec 30;15(1):28. doi: 10.3390/toxins15010028 (PMC9866706; doi:10.3390/toxins15010028)
Supplement: Supplementary file 1 [file toxins-15-00028-s001.zip › toxins-2097823-supplementary.pdf]

toxins

MDPI

Table S1. MALDI-TOF characterization of novel nAChR-targeting toxins purified from *Metridium senile* venom and their established N-terminal sequences (cysteine residues are shown with bold).

Peptide name	Average MW measured by MALDI-TOF (Da)	Calculated average MW of the respective predicted target peptides deduced from cDNAs after proposed posttranslational modifications (Da)	N-terminal sequence established by Edman degradation
**α-AnmTX-Ms11a-1** (short name Ms11a-1)	4594.8	4594.38	G**C**KKLNSN**C**TRQYRE**CC**HGLV**C**RRPNYGNGRGILWR
**α-AnmTX-Ms11a-2** (Ms11a-2)	4653.4	4653.41	G**C**KNLNSH**C**YRQHRE**CC**HGLV**C**R
**α-AnmTX-Ms11a-3** (Ms11a-3)	4918.4	4918.82	G**C**KKLNSY**C**TRQHRE**CC**HGLV**C**RRPDYGIG
**α-AnmTX-Ms11a-4** (Ms11a-4)	3274.8	3274.79	**C**AQTGGT**C**SKSKD**CC**IVTAI**C**STA

(a) Ms11a-1

DNA: gattcgggtgtgaacatcaccaagatagcaacgtttcaagcttcacactcaaccgaactg

+1fr: ·D··S··G··V··N··I··T··K··I··A··T··F··Q··A··S··H··S··T··E··L·

DNA: gatattacactcaaccgaactggatattactattataagggattcgaa**atg**gcttcaaag

+1fr: ·D··I··T··L··N··R··T··G··Y··Y··Y··Y··K··G··F··E··M··A··S··K·

DNA: attttctttgtccttgcagtgtttcttgtgatgtcggctgtgctccccgaatctttcgcg

+1fr: ·I··F··F··V··L··A··V··F··L··V··M··S··A··V··L··P··E··S··F··A·

DNA: ggctgcaagaaattgaattcgaattgttcgagacaataccgtgagtgttgtcatggtttg

+1fr: ·**G··C··K··K··L··N··S··N··C··S··R··Q··Y··R··E··C··C··H··G··L**·

DNA: gtgtgcagaaggcctaactatggcaatggcagagggattctttggagatgtgtcaaggcc

+1fr: ·**V··C··R··R··P··N··Y··G··N··G··R··G··I··L··W··R··C··V··K··A**·

DNA: aagaagtgaatggatgcttataataaaggataagctcttcattggttaagaataatacta

+1fr: ·***K··K***··*··M··D··A··Y··N··K··G··*··A··L··H··W··L··R··I··I··L·

DNA: atactaatactaatgtttttcaggaggttgctctaaagaactaaaattcaaactgtatta

+1fr: ·I··L··I··L··M··F··F··R··R··L··L··*··R··T··K··I··Q··T··V··L·

DNA: acactgtaattcaaaaaggaaataaactatcatagttgattcaaaaaaaaaaaaaaaaaa

+1fr: ·T··L··*··F··K··K··E··I··N··Y··H··S··*··F··K··K··K··K··K··K·

(b) Ms11a-4

DNA: agatacaagtgaacattgaagtcgttcttgcaagtgaatctctcgagcttgataacc**atg**

+1fr: ·R··Y··K··*··T··L··K··S··F··L··Q··V··N··L··S··S··L··I··T··M·

DNA: aaggtccttgttgcagtccttgtctttgctctactgatgtgtatgtttgttgacatcgcg

+1fr: ·K··V··L··V··A··V··L··V··F··A··L··L··M··C··M··F··V··D··I··A·

DNA: gaaagtcgtcgtcgagacaacccagaataccccagtgggcttcgatatgacgaggagatg

+1fr: ·E··S··*R··R··R··D··N··P··E··Y··P··S··G··L··R··Y··D··E··E··M*·

DNA: ggtgttttcaaacggtgcgctcaaacgggagggacatgctccaaatctaaagattgctgt

+1fr: ·*G··V··F··K··R*··**C··A··Q··T··G··G··T··C··S··K··S··K··D··C··C**·

DNA: attgtaacagctatctgcagtacagcaacaagtcctaaaacatgtttttatggctagtga

+1fr: ·**I··V··T··A··I··C··S··T··A··T··S··P··K··T··C··F··Y**··***G***··*··*·

```
DNA:  aagagcgggaatatagtgtgtgcatgcagattttacaattgaagatgtctttcacttagc
+1fr: ·K··S··G··N··I··V··C··A··C··R··F··Y··N··*··R··C··L··S··L··S·

DNA:  tggaatcatgtaaaataaaaccactctttgcttgtaatggtgatcaggattattataata
+1fr: ·W··N··H··V··K··*··N··H··S··L··L··V··M··V··I··R··I··I··I··I·

DNA:  aaacatgtaatggtgtcga
+1fr: ·K··H··V··M··V··S·
```

Figure S1. Structural organization of precursor proteins. ***(a)*** nucleotide sequence of *ms11.1* cDNA accompanied by deduced amino acid sequence. **(b)** nucleotide sequence of *ms11.4* cDNA accompanied by deduced amino acid sequence. The signal peptide sequences are *underlined*, determined mature peptide sequences are bold and highlighted gray, and the propeptide sequences are shown in italics.

Table S2. 3D-alignment of Ms11a-3 with proteins in PDB database.

<table>
<tr><td>№</td><td>PDB ID (Ref.)</td><td>Name</td><td>Q-score (RMSD)</td><td>N_{align} (N_{all})</td><td>Source organism</td><td>Target (that was in the corresponding Ref.)</td><td>No (or weak) effect</td></tr>
<tr><td>1</td><td>2MXM (doi.org/10.1124/mol.115.098178)</td><td>TRTX-Tp1a</td><td>0.66 (1.07Å)</td><td>32(33)</td><td>Thrixopelma pruriens</td><td>hNa$_v$1.1 (IC$_{50}$ 101±30 nM);
hNa$_v$1.3 (IC$_{50}$ 1.3±4.7 nM);
hNa$_v$1.5 (IC$_{50}$ >500 nM);
hNa$_v$1.7 (IC$_{50}$ 2.1±1.3 nM)</td><td>human voltage-gated calcium channels (5 μM);
nAChR (5 μM)</td></tr>
<tr><td colspan="8">10 20 30 40
Ms11a-3:GCKKLNSYCTRNHRECCHGLVCR-RPDYGIGRGILWKCTRARK
2MXM :DCLKFGWKCNPRNDKCCSGLKCGSNH---------NWCKLHL-
10 20 30</td></tr>
<tr><td>2</td><td>2MQF (doi.org/10.1016/S0014-5793(03)01303-6; doi.org/10.1124/mol.115.100784)</td><td>TRTX-HHN2B</td><td>0.64 (1.06Å)</td><td>32(34)</td><td>Haplopelma hainanum</td><td>para/tipE (IC$_{50}$ 4.3±0.3 μM)
rNa$_v$1.2/β$_1$
(IC$_{50}$ 68±6 μM)</td><td></td></tr>
<tr><td colspan="8">10 20 30 40
Ms11a-3:-GCKKLNSYCTRNHRECCHGLVCR-RPDYGIGRGILWKCTRARK
2MQF :AECKGFGKSCVPGKNECCSGYACNSRD---------KWCKVLL-
10 20 30</td></tr>
<tr><td>3</td><td>6BR0 (doi.org/10.1074/jbc.RA118.002553)</td><td>CCOTX-I</td><td>0.63 (1.26Å)</td><td>32(33)</td><td>Ceratogyrus marshalli</td><td>hNa$_v$1.2 (EC$_{50}$ 6.1±0.7 nM);
hNa$_v$1.4 (EC$_{50}$ 263±45 nM);
hNa$_v$1.5 (EC$_{50}$ 188±20 nM);
hNa$_v$1.6 (EC$_{50}$ 40.6±5.8 nM);
hNa$_v$1.7 (IC$_{50}$ 129.1±6.5 nM)</td><td></td></tr>
<tr><td colspan="8">10 20 30 40
Ms11a-3:GCKKLNSYCTRNHRECCHGLVCR-RPDYGIGRGILWKCTRARK
6BR0 :DCLGWFKSCDPKNDKCCKNYTCSRRD---------RWCKYDL-
10 20 30</td></tr>
<tr><td>4</td><td>1MB6 (doi.org/10.1074/jbc.M204063200)</td><td>HWTX-IV</td><td>0.59 (0.95Å)</td><td>31(35)</td><td>Haplopelma schmidti</td><td>neuronal tetrodotoxin-sensitive voltage-gated sodium channel
of adult rat dorsal root ganglion neurons (IC$_{50}$ 30 nM)</td><td>tetrodotoxin-resistant voltage-gated sodium channel</td></tr>
</table>

10 20 30 40<br>Ms11a-3:GCKKLNSYCTRNHRECCH--GLVCRRPDYGIGRGILWKCTRARK<br>1MB6 :ECLEIFKACNPSNDNCCKSSKLVCSRK--------TRWCKYNI-<br>10 20 30							
5	2MPQ (doi.org/10.1111/bph.13081)	TRTX-Hd1a	0.59 (1.13Å)	32(36)	*Haplopelma doriae*	hNa$_v$, especially hNa$_v$1.1 and hNa$_v$1.7	hERG
10 20 30 40<br>Ms11a-3:-GCKKLNSYCTRNHRECCH--GLVCR-RPDYGIGRGILWKCTRARK<br>2MPQ :GACLGFGKSCNPSNDNCCKSSSLACSTKH---------KWCKYEL-<br>10 20 30							
6	6GFT (doi.org/10.1111/bph.14628)	CyrTx-1a	0.58 (1.07Å)	30(33)	*Cyriopagopus schioedtei*	hNa$_v$1.1 (IC$_{50}$ 72±10 nM);<br>hNa$_v$1.2 (IC$_{50}$ 75.5±4.3 nM);<br>hNa$_v$1.3 (IC$_{50}$ 307±15 nM);<br>hNa$_v$1.6 (IC$_{50}$ 115±7.5 nM);<br>hNa$_v$1.7 (IC$_{50}$ 130±2 nM);<br><br>hNa$_v$1.4 (IC$_{50}$ 7.7±0.2 μM);<br>hNa$_v$1.5 (IC$_{50}$ >10μM);<br>hNa$_v$1.8 (IC$_{50}$ >10 μM);	hCa$_v$1.2, 3.1, 3.2<br>hK$_v$7.1, 11.1<br>hK$_{ir}$2.1
10 20 30 40<br>Ms11a-3:GCKKLNSYC-TRNHR-ECCHGLVCR-RPDYGIGRGILWKCTRARK<br>6GFT :ECKGFGKSCVP--GKNECCSGLTCSNKH---------KWCKVLL-<br>10 20 30							
7	2N1N (doi.org/10.1038/nature01473)	VSTx1	0.56 (1.72Å)	33(35)	*Grammostola rosea*	K$_V$AP	
10 20 30 40<br>Ms11a-3:-GCKKLNSYCTRNHRECCHGLVCRRPDYGIGRGILWKCTRARK-<br>2N1N :SECGKFMWKCKN-SNDCCKDLVCSSRW--------KWCVLASPF<br>10 20 30							
8	1EIT (doi.org/10.1021/bi952605r)	μ-Aga-I	0.55 (1.65Å)	33(36)	*Agelenopsis aperta*	Voltage-gated sodium channels	
10 20 30 40<br>Ms11a-3:GCKKLNSYCTRNH-RECCHGLVCRRPDYGIGRGILWKCTRARK<br>1EIT :ECVPENGHCRDWYDE-CCEGFYCSCRN-----PPKCICRNNN-<br>10 20 30							

9	2M9L (doi.org/10.1016/j.cub.2014.01.013; doi.org/10.1016/j.toxicon.2006.09.014)	ProTx-I	0.54 (1.37Å)	31(35)	*Thrixopelma pruriens*	TRPA1 (IC_{50} 389±77 nM); $Na_v1.2$, $Na_v1.5$, $Na_v1.7$, $Na_v1.8$	
10 20 30 40<br>Ms11a-3:GCKKLNSYCTRNHRECCHGLVCR-RPDYGIGRGILWKCTRARK--<br>2M9L :ECRYWLGGCS-AGNTCCKHLVCSRRH---------GWCVWDGTFS<br>10 20 30							
10	1LU8 (doi.org/10.1016/j.toxicon.2006.09.030)	GsMTx-4	0.54 (1.72Å)	32(34)	*Grammostola rosea*	TRPC1	voltage-gated channels
10 20 30 40<br>Ms11a-3:GCKKLNSYCTRNHRECC-HGLVCRRPDYGIGRGILWKCTRARK<br>1LU8 :GCLEFWWKCNPNDDKCCRPKLKCSKLF--------KLCNFSF-<br>10 20 30							
11	7A64 (doi.org/10.1021/acsptsci.1c00072)	Tsp1a	0.53 (1.55Å)	28(28)	*Thrixopelma sp.*	hNaV1.7 (IC_{50} 10 nM); hNaV1.1 (IC_{50} 450 nM); hNaV1.2 ((IC_{50} 250 nM)	
10 20 30 40<br>Ms11a-3:GCKKLNSYCTRNHRECCHGLVCRRPDYGIGRGILWKCTRARK<br>7A64 :YCNKFLWTCD-SERPCCEGLVCRL-----------WCKIN--<br>10 20							

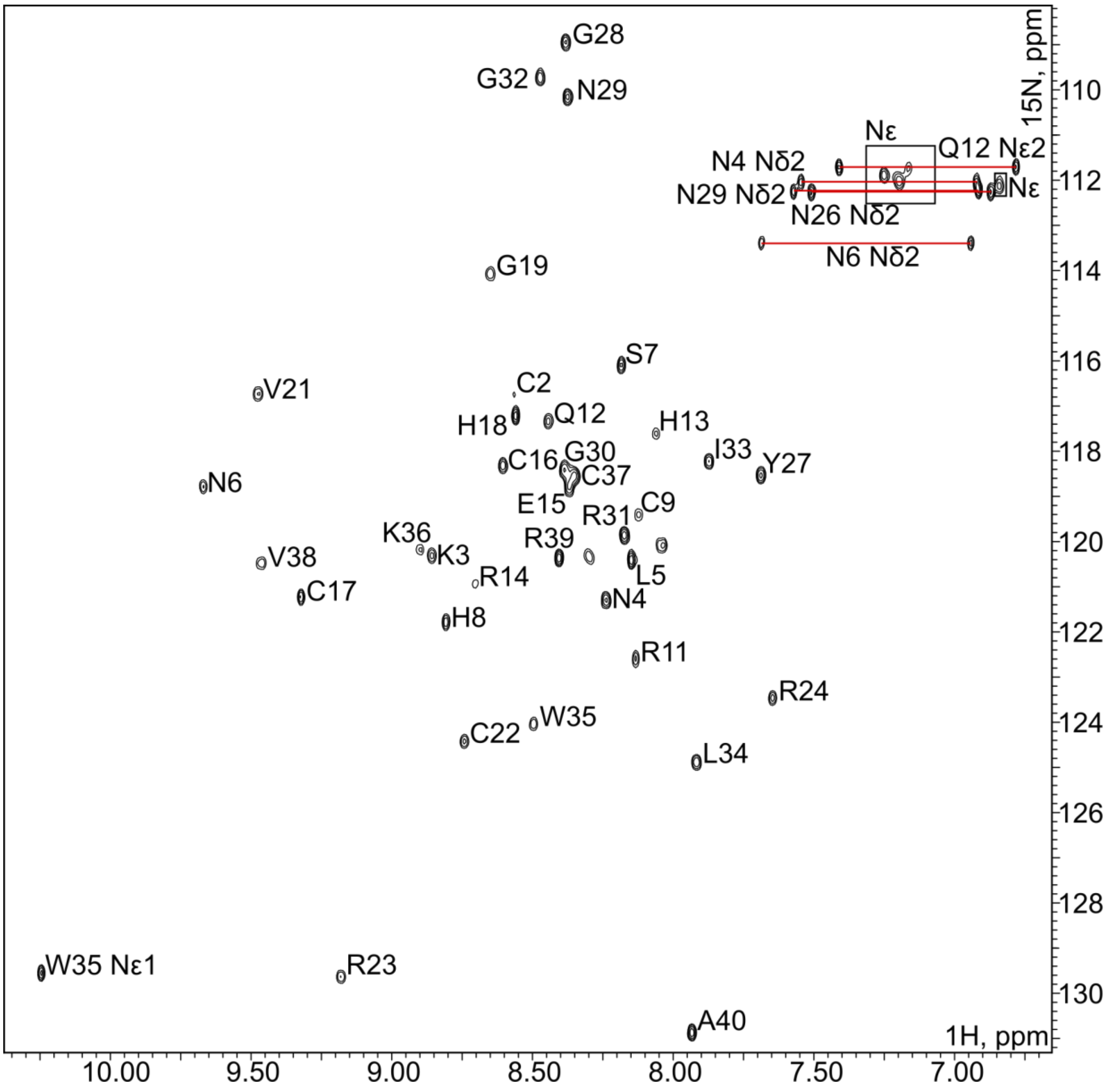

Figure S2. $^{1}H^{15}N$-HSQC spectrum of Ms11a-2. Spectrum was recorded at 30 °C, pH 5.2. The assignment of cross-peaks is indicated.

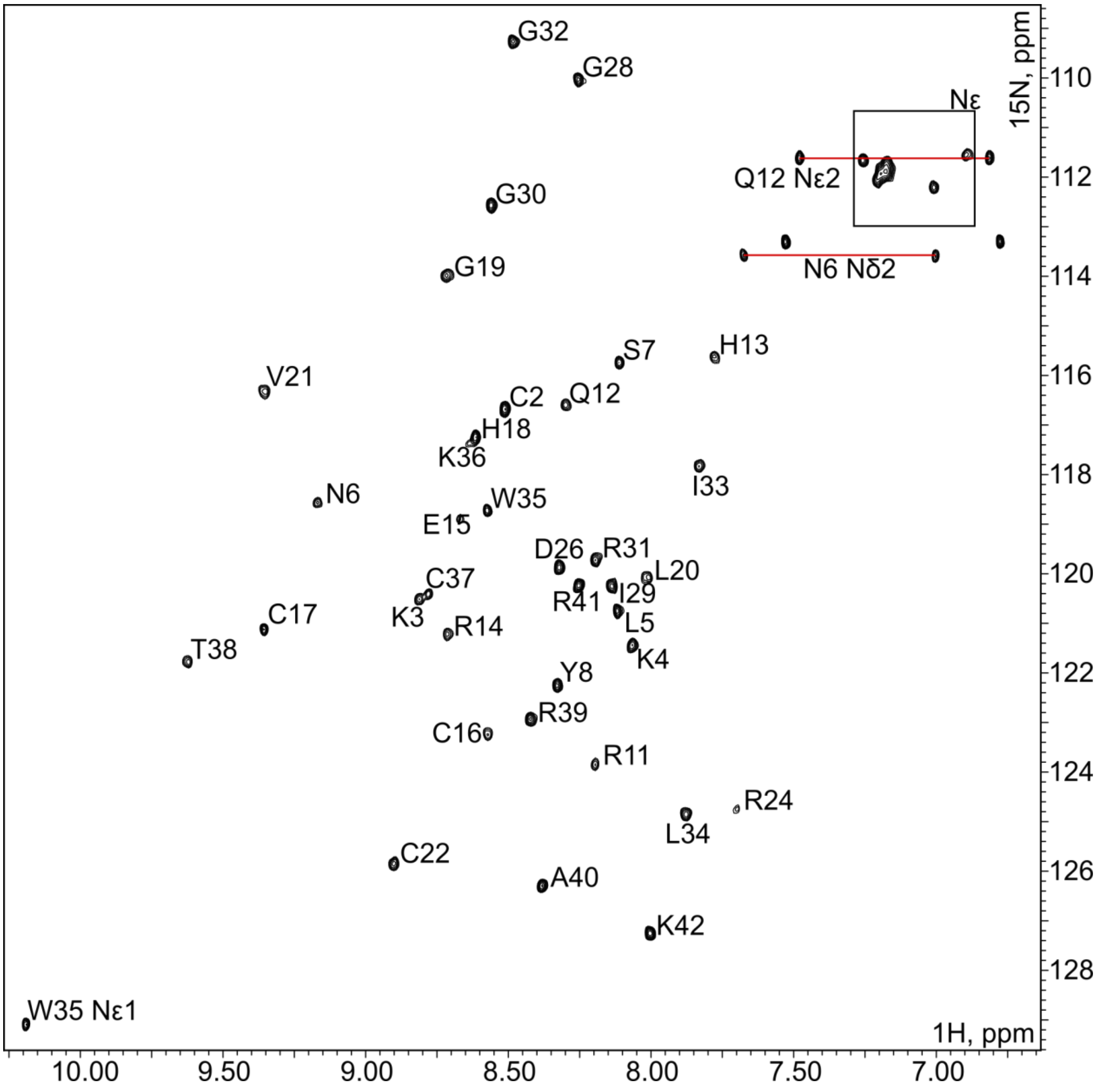

Figure S3. $^{1}H^{15}N$-HSQC spectrum of Ms11a-3. Spectrum was recorded at 30 °C, pH 4.5. The assignment of cross-peaks is indicated.
